# Management of Impacted Premolar With Root Dilaceration Caused by Radicular Cyst in Deciduous Molar: A Case Report

**DOI:** 10.1155/crid/8219795

**Published:** 2025-06-26

**Authors:** Shuhao Xu, Xiaolong Li, Yu Zhang, Wei Li

**Affiliations:** ^1^Department of Stomatology, Deyang People's Hospital, Deyang, China; ^2^Department of Respiratory and Critical Care Medicine, Deyang People's Hospital, Deyang, China

**Keywords:** case report, impacted teeth, orthodontic traction, primary molar, radicular cysts, root dilaceration

## Abstract

Radicular cyst is the most common inflammatory odontogenic cyst in the oral cavity, but it rarely occurs in deciduous teeth. Radicular cysts in deciduous teeth can lead to root dilaceration in permanent successor teeth, resulting in impacted permanent teeth. We presented an 11-year-old boy who underwent decompression and drainage of a radicular cyst in deciduous tooth, followed by early orthodontic traction of permanent successor tooth with root dilaceration. The cyst healed successfully, and the impacted premolar was gradually aligned into the dental arch with favorable root development This case demonstrates that timely decompression of radicular cysts in deciduous teeth can effectively protect the successor permanent teeth. Meanwhile, for cases where permanent tooth impaction with root dilaceration has already occurred, timely orthodontic traction can move the root from the bone cortex into the cancellous bone to obtain growth space.

## 1. Introduction

Radicular cysts are the most common inflammatory odontogenic cysts in the oral cavity, arising from epithelial rests of Malassez in response to pulp necrosis, but they are rare in deciduous teeth, accounting for only 0.5%–3.3% of all radicular cysts in both deciduous and permanent teeth [1]. Radicular cysts in deciduous teeth are more likely associated with the mandibular primary molars [2], commonly caused by dental pulp necrosis due to caries or trauma, which may affect the development and normal eruption of permanent successor teeth [3]. Cyst formation in pediatric cases can lead to bone expansion, resorption, delayed tooth eruption, malposition, enamel defects, and damage to developing permanent teeth [4]. In severe cases, it can cause root dilaceration of permanent successor teeth, leading to impaction of those teeth [5]. But unfortunately, radicular cysts are usually asymptomatic unless secondarily infected [6]. When swelling, tooth displacement, and delayed eruption of permanent teeth are observable, the lesion has already clearly formed [7]. Radicular cysts of deciduous teeth are often detected on radiographic examination, presenting as round, unilocular, radiolucent lesions around the root apex.

Usually, there are three ways to conduct to treat radicular cysts—surgical enucleation, marsupialization, or decompression [8]. In cases of radicular cysts in deciduous teeth, permanent tooth germs should be preserved whenever possible. Extraction of the affected deciduous teeth often allows smaller radicular cysts to resolve spontaneously [9]. However, large radicular cysts can be decompressed. The advantage of decompression reveals in preservation of unerupted successors, avoidance of surgical damage to closely adjacent anatomical structures, maintenance of oral tissues, and minimal impairment of bone growth [10].

For impacted permanent teeth or root dilaceration, orthodontic traction is carried out at the early stage to induce normal eruption of permanent teeth and maintain the integrity of the dentition. Previous studies have indicated that early release of the impacted state by orthodontic traction would aid the root development of impacted teeth in terms of root growth and total root length [11].

In this case report, we presented an 11-year-old boy who underwent decompression and drainage of a radicular cyst in deciduous tooth, followed by early orthodontic traction of permanent successor tooth with root dilaceration. The cyst healed successfully, and the impacted premolar was gradually aligned into the dental arch with favorable root development.

## 2. Case Presentation

An 11-year-old boy went to our outpatient clinic with a complaint of “swelling and pain in the gums of the left lower posterior tooth for 1 week.” He was healthy, with normal general development, and denied a history of systemic diseases, family diseases, and drug allergies. The left lower deciduous tooth had a history of filling treatment. Extraoral examination showed the bilateral maxillofacial regions were symmetrical, without obvious maxillofacial swelling. No swollen lymph nodes were palpated in the left submandibular region, and mandibular function movement was not limited. Intraoral examination showed fillings were observed on the distal and middle adjacent surface of Tooth 74, with painful knocking (+) and loose (I), and the gingiva in the buccal apical area was swollen and tough.

**Figure 1:**
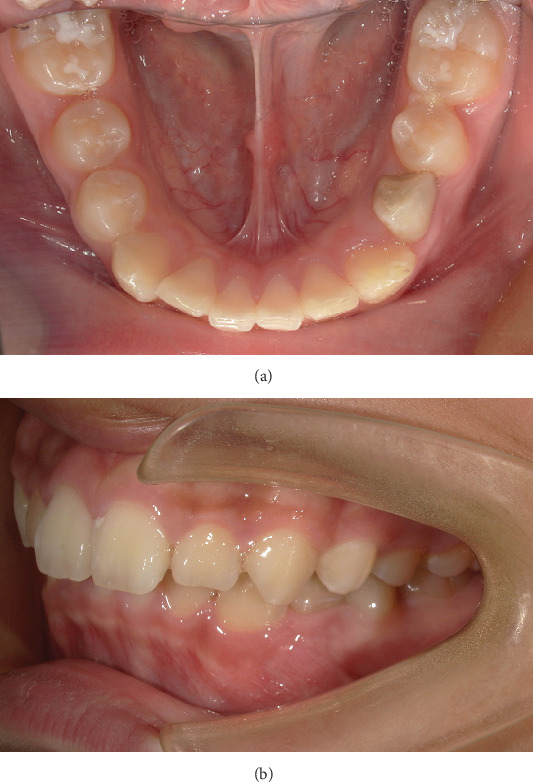
(a, b) Pretreatment intraoral photographs.

Intraoral photographs show that he presented with mixed dentition. Tooth 74 has not been replaced, and the remaining deciduous teeth have been replaced. Fillings were observed on the distal and middle adjacent surface of Tooth 74, and the gingiva in the buccal apical area was swollen and tough (Figure 1).

**Figure 2:**
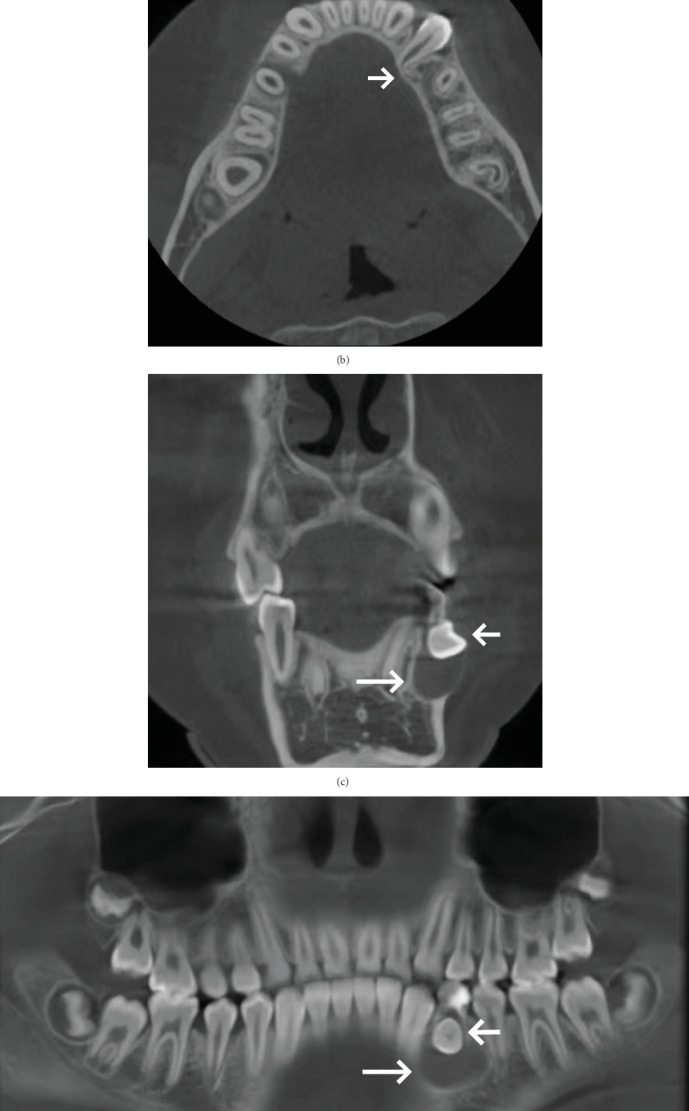
(a–e) Pretreatment CBCT examination: CBCT showed Tooth 74 apical areas with an oval transmission of about 11 × 7 mm (long arrow), the germ of permanent Tooth 34 was inclined in the buccal direction, impacted horizontally, and the root was curved (short arrow).

Cone beam computed tomography (CBCT) showed Tooth 74 apical areas with an oval transmission of about 11 × 7 mm. The germ of the permanent Tooth 34 was inclined in the buccal direction, impacted horizontally, and the root was curved. The roots developed to the Nolla Stage 8 (Figure 2).

Based on the medical history, clinical examination, and CBCT findings, the diagnosis we considered was (1) radicular cyst of Tooth 74 and (2) impacted Tooth 34 with root dilaceration. In this case, the radicular cyst of Tooth 74 required differential diagnosis from other kinds of odontogenic cysts. Considering the history of caries restoration in Tooth 74 and the oval radiolucency observed around its apical region on CBCT, a diagnosis of radicular cyst associated with Tooth 74 is proposed.

## 3. Treatment Progress

After critical evaluations, we proceed a three-step treatment plan: (1) decompression and drainage of the radicular cyst in Region 74, (2) orthodontic traction of Tooth 34, and (3) in the later stage, the need for Stage II comprehensive orthodontic treatment should be evaluated according to the requirements and the occlusion of the child.

**Figure 3:**
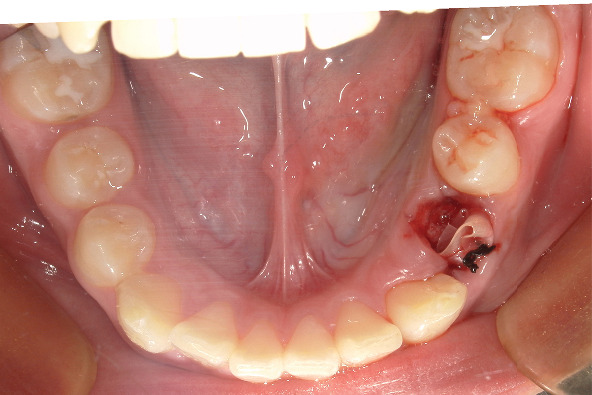
Intraoral photograph: pulling out 74, flushing the capsule cavity, and reaching the drainage strip.

Local infiltration anesthesia was performed, and the tooth was pulled out following satisfactory anesthesia. The cyst wall was explored through the tooth extraction wound, and purulent exudation was observed after puncturing the cyst wall. A drainage strip was placed after repeated flushing with normal saline until the flushing solution became clear. At the same time, to prevent the drainage strip from falling off and being swallowed or aspirated by the child, the rubber drainage strip was sutured using nonabsorbable suture material (nylon filament, 4-0) (Figure 3).

**Figure 4:**
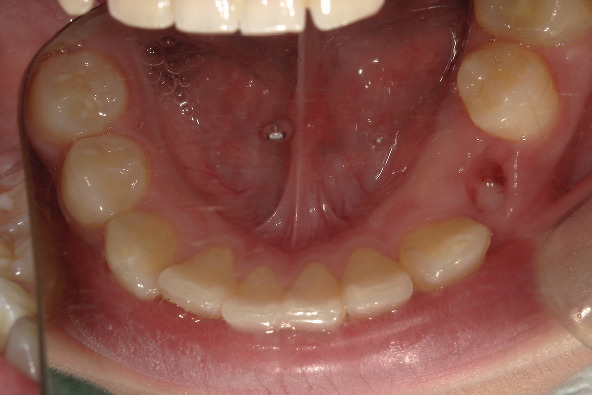
Intraoral photographs: 1 week after the operation.

After 1 week, the drainage strip was removed and washed continuously. No purulent exudation was observed. At this time, the crown of Tooth 34 was visible from the extraction wound of Tooth 74 (Figure 4).

**Figure 5:**
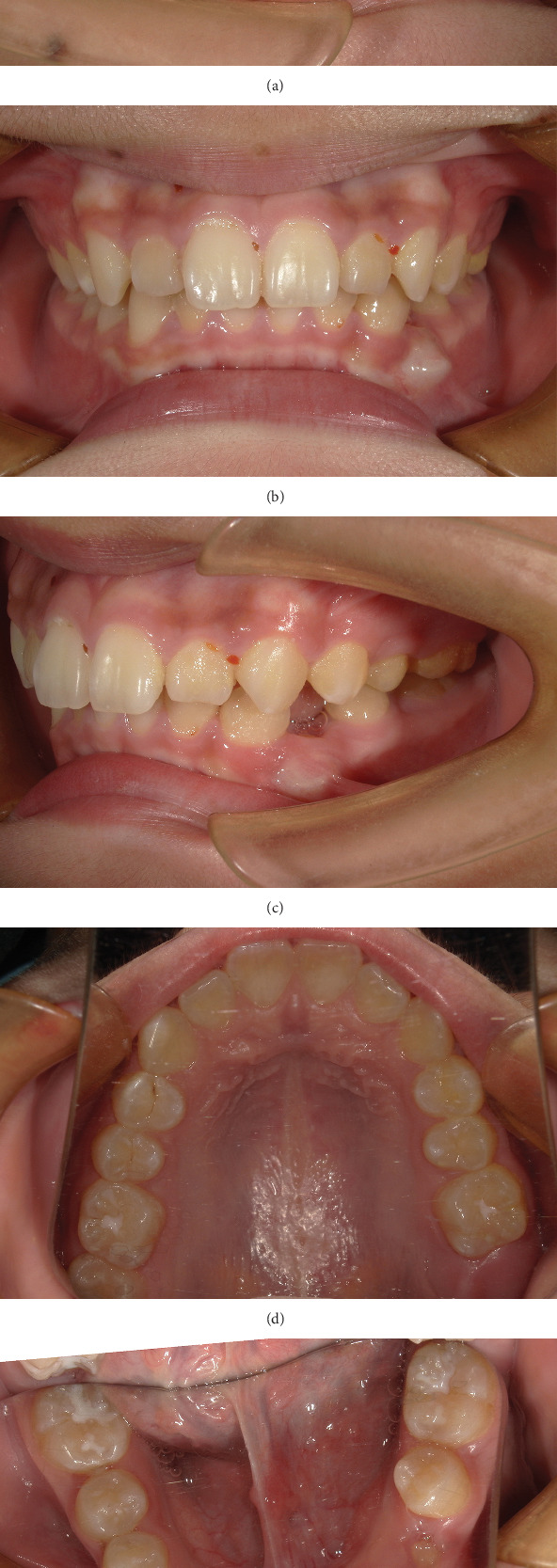
(a–e) Intraoral photographs: follow-up 2 months after the operation.

After 2 months of follow-up, Tooth 74 extraction wound healed well, with Tooth 34 crown visible, and Tooth 34 crown swelled palpably in the buccal gingiva (Figure 5).

**Figure 6:**
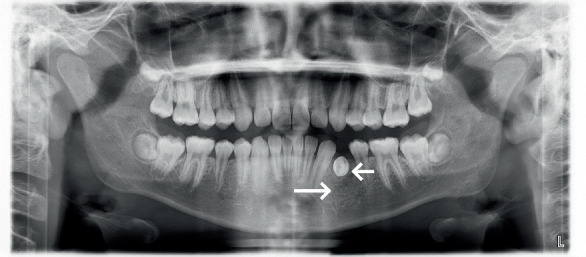
Panoramic radiograph: 2 months after operation. The panoramic radiograph showed that the transmission shadow of the apical area disappeared, bone mineral density increased (long arrow), and the buccal–lingual impacted image in Tooth 34 (short arrow).

Two months after the operation, the panoramic radiograph showed that the transmission shadow of the apical area disappeared bone mineral density increased and the buccal–lingual impacted image in Tooth 34 (Figure 6).

His parents were informed that the radicular cyst in deciduous teeth was healing well, and a plan was made to perform orthodontic traction of Tooth 34. His parents provided informed consent for the follow-up treatment plan and signed the consent form.

**Figure 7:**
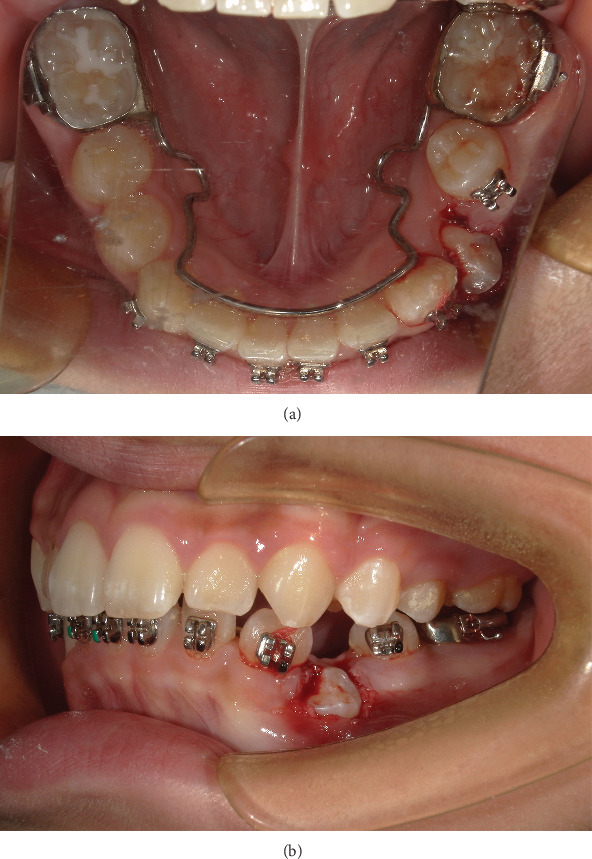
(a, b) Intraoral photographs: at the beginning of exposing and orthodontic traction 34.

A mandibular lingual arch was made and bonded to strengthen anchorage, maintain the mandibular arch, and support the bonding of a local fixed orthodontic appliance (HX metal brackets, Shinye Biotech, China). After the contraindication was eliminated, fenestration was performed on Tooth 34. The crown of Tooth 34 was exposed, and local compression was applied to effectively control the bleeding (Figure 7).

**Figure 8:**
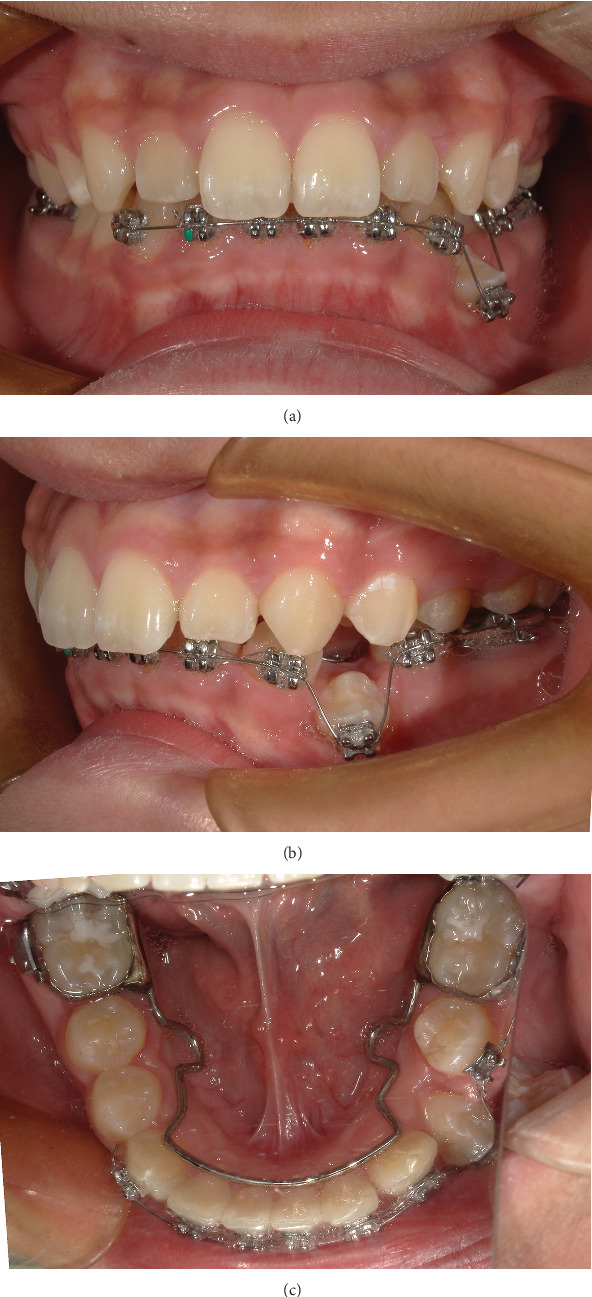
(a–c) Intraoral photographs: 1 week after exposure.

One week after decompression and following wound healing, the bracket of Tooth 34 was bonded and drawn with 0.012-in. hyperelastic NITI wire (IMD Materials Technology Co., China) (Figure 8).

**Figure 9:**
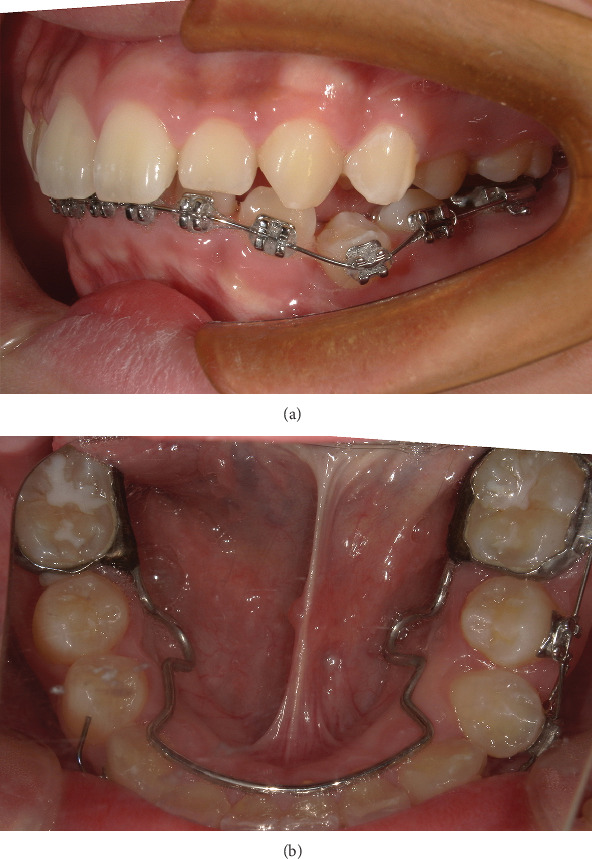
(a, b) Intraoral photographs 2 months after exposing 34.

Two months after decompression, the bracket of Tooth 34 was gradually aligned into the dental arch and was replaced with 0.018-in. NITI wire (IMD Materials Technology Co., China) to continue to align the teeth (Figure 9).

## 4. Results

Throughout the entire decompression and orthodontic traction treatment process, this child and his parents cooperated exceptionally well. Apart from experiencing minor pain on the day following the decompression surgery and mild discomfort during orthodontic procedures, no other significant complaints of discomfort were reported.

**Figure 10:**
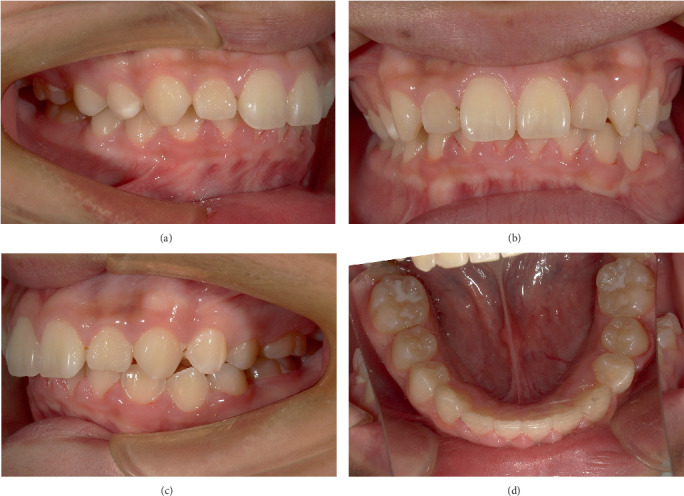
(a–d) Intraoral photographs: 6 months after exposure.

**Figure 11:**
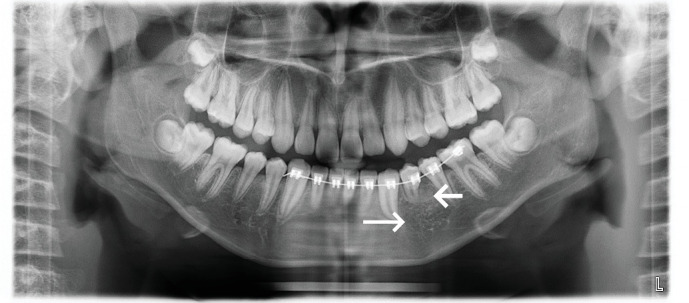
Panoramic radiograph was reexamined 6 months after exposure: panoramic radiograph showed that Tooth 34 had curved roots (short arrow), without obvious abnormality in the bone mineral density at the apical area (long arrow).

Six months after decompression, Tooth 34 was aligned into the dental arch (Figure 10), and Tooth 34 had normal pulp vitality, without abnormal looseness. His parents were satisfied with the treatment outcome at this stage. Due to the academic reasons of their child, they did not consider Stage II orthodontic comprehensive treatment for the time being and requested the removal of the fixed appliance from the lower half of the mouth, thereby ending treatment at this stage and using Hawley retainer for the passive maintenance. A panoramic radiograph was taken before removing the fixed appliance, and it showed that Tooth 34 had curved root, without obvious abnormality in the bone mineral density at the apical area (Figure 11).

The treatment achieved satisfactory results as reported by both the patient and parents. We instructed the patient to maintain optimal oral hygiene at home, consistently wear the Hawley retainer as prescribed, and return for follow-up visits every 3–6 months.

## 5. Discussion

The radicular cyst in deciduous teeth is classified as an inflammatory odontogenic cyst, typically resulting from dental pulp necrosis caused by caries, trauma, abnormal tooth development, and other factors [3]. It can also arise from the use of materials containing cresol in dental pulp treatment. The cresol, upon binding with tissue proteins, exhibits antigenic properties and has been demonstrated to trigger both humoral and cell-mediated immune responses [4]. Radicular cysts in deciduous teeth can cause local pain and swelling, as well as apical bone destruction. In severe cases, it may lead to extensive bone destruction and affect the inherited permanent tooth germ, resulting in impaction of the permanent successor teeth. Viggness et al. [12] indicated that radicular cyst can be better radiologically evaluated three-dimensionally with the help of CBCT than conventional intraoral periapical radiographs, which do not reveal the exact side of perforation of the cortical plate caused by expansion of the cyst.

In recent years, several scholars have published case reports on radicular cysts of deciduous teeth. We have summarized and compared these cases (Table 1). As demonstrated in these reported cases, small radicular cysts in deciduous teeth often resolve spontaneously after extraction of the affected deciduous teeth. For extensive radicular cysts in deciduous teeth, permanent tooth germs should be preserved as much as possible, because children have a strong bone regeneration ability and can repair bone defects quickly after operation [9]. Therefore, for a large range of radicular cysts in deciduous teeth, relatively conservative treatment schemes, such as marsupialization or decompression, should be considered to protect the permanent tooth germs under deciduous teeth as much as possible. Marsupialization is a surgical technique involving the fenestration of the cystic wall by suturing the cystic lining to the oral mucosa, thus connecting the cystic cavity to the oral cavity [9]. The success of marsupialization depends on operator's surgical technique, good cooperation from both the child and parents, proper home care and irrigation, and regular follow-up visits. Decompression which only requires a much smaller opening in the cystic wall has been consistently and effectively applied in the treatment of various types of odontogenic cysts. Previous studies proposed a reduction of the intraluminal pressure in cysts made by decompression restores the original anatomy by the surrounding tissues, like bone and periost [8]. The advantage of decompression reveals in preservation of unerupted successors, avoidance of surgical damage to closely adjacent anatomical structures, maintenance of oral tissues, and minimal impairment of bone growth [22]. Therefore, we opted for decompression—a minimally invasive and technically straightforward approach—to manage the radicular cyst in the deciduous tooth, with the primary objective of preserving the permanent successor tooth.

Root dilaceration refers to abnormal tooth development in which the crown or root deviates from the long axis of the tooth, often resulting in a certain bending angle between the crown and root (or part of the root), and this condition is considered an abnormality in tooth morphology [23]. Root dilaceration usually fails to erupt smoothly, resulting in impaction. Impacted teeth with root dilaceration are usually caused by acute mechanical injury or developmental interference factors, including apical periodontitis of deciduous teeth, cleft lip and palate, ectopic tooth germ development, soft tissue scarring, insufficient space, or interference from surrounding structures, dental tumor, dental follicles, adhesion of the deciduous tooth root, genetic factors, and some syndromes [24, 25]. This case demonstrates root dilaceration and impaction of the permanent successor teeth caused by a radicular cyst in the deciduous teeth. It highlights the importance of maintaining the health of deciduous teeth for the proper development and eruption of permanent teeth. The prevention and treatment of children's dental diseases still need continued efforts. For impacted teeth without root dilaceration, early orthodontic traction can reduce the risk of developing root dilaceration. For the affected teeth with root dilaceration, early orthodontic traction can use the development potential of the epithelial root sheath to produce a secondary curvature, thereby promoting continued root development. It increases the root length and reduces the crown–root angle [26, 27]. At the same time, orthodontic traction can prevent alveolar bone atrophy and adjacent tooth inclination after missing teeth. Even if the tooth becomes loose over time due to excessive chewing forces, it preserves sufficient alveolar bone mass for adult implant restoration.

Therefore, early detection, diagnosis, and traction are needed for impacted teeth with root dilaceration. Moreover, even for root dilaceration with short roots or bone fenestration after traction, as long as there is no significant abnormality in pulp vitality or abnormal looseness, the long-term postoperative effect of traction treatment is ideal. This may be attributed to the favorable physical chimerism between the curved roots and the alveolar bone, allowing the crown to withstand normal bite force [28, 29].

This case report has certain limitations. Notably, histological biopsy was omitted based on the conclusive clinical and radiographic findings, while also reducing financial burden for the patient's family. Additionally, the follow-up period in this case was relatively short, limiting the ability to effectively assess long-term prognosis. Cases where radicular cysts of deciduous teeth cause root dilaceration and impaction of permanent successor teeth remain particularly challenging to manage.

## 6. Conclusion

Early detection, diagnosis, and intervention should be carried out for radicular cysts in deciduous teeth. For larger radicular cysts in deciduous teeth, decompression is an effective treatment method that can also protect the permanent successor teeth. For permanent successor tooth impaction or even root dilaceration, early orthodontic traction should be performed to move the root from the bone cortex into the cancellous bone to obtain growth space. This process can increase the root length and reduce the crown–root angle. Even for curved teeth with short roots or bone fenestration after traction, the long-term postoperative effect of traction treatment may be ideal.

## Figures and Tables

**Table 1:** List of papers reporting radicular cysts of deciduous teeth in recent years.

**Author**	**Year**	**Patient's characteristic**	**Affected tooth**	**Cyst size**	**History**	**Treatment options**
Takaaki et al. [13]	2024	7-year-old girl	84, 85	No description available	Stainless steel crown restoration of 85	Surgical enucleation
Misa et al. [1]	2024	6-year-old boy	72, 73	Diameter of approximately 16 mm	No significant findings	Surgical enucleation
Raju et al. [14]	2024	6-year-old boy	74, 75	15 × 25 mm	Pulpotomy of 74	Surgical enucleation
Izzetti et al. [15]	2024	11-year-old boy	84	No description available	Endodontic treatment	Marsupialization
Anvika et al. [16]	2023	6-year-old girl	54, 55	20 × 30 mm	No significant findings	Surgical enucleation
Tanzeem et al. [17]	2022	7-year-old boy	74, 75	15 × 20 mm	Pulpectomy of 74 and pulpotomy of 75	Marsupialization
Tanzeem et al. [17]	2022	8-year-old girl	84	20 × 20 mm	Caries	Marsupialization
Shweta et al. [18]	2021	5-year-old girl	85	20 × 30 mm	Trauma to the jaws, caries	Surgical enucleation
Manjaree et al. [4]	2020	7-year-old boy	74	50 × 80 mm	Caries	Surgical enucleation
Damian et al. [19]	2020	5-year-old boy	54, 55	No description available	Restoration	Surgical enucleation
Vo et al. [20]	2019	5-year-old girl	64	Diameter of over 30 mm	Pulp therapy with gutta percha	Marsupialization
Orlando et al. [9]	2019	9-year-old boy	64, 65	No description available	Caries	Marsupialization
Shrirang et al. [21]	2018	5-year-old boy	85	15 × 20 mm	Caries	Surgical enucleation

## Data Availability

The data that support the findings of this study are available from the corresponding author upon reasonable request.
